# Editorial: The forever young Na^+^/H^+^ exchanger family: New insights in its structure, function and regulation

**DOI:** 10.3389/fphys.2022.1044669

**Published:** 2022-10-18

**Authors:** Francesca Di Sole, Victor Babich

**Affiliations:** ^1^ Department of Physiology and Pharmacology, Des Moines University, Des Moines, IA, United States; ^2^ Mercy College of Health Sciences, Des Moines, IA, United States

**Keywords:** Na/H exchanger, hypertension, diarrhea, microbiota, cell volume, pH

The Na^+^/H^+^ exchangers (NHEs) are a group of ion transporter proteins with an essential role in regulation of cytoplasmic and organellar pH and cell volume. NHEs are well-known for their roles in human physiology and disease *via* regulation of systemic acid-base and volume homeostasis. This Research Topic provided a comprehensive update on NHE research subjects and view of directions the field is moving in the future. These goals were achieved by attracting expert contributors who have given a prospective on how recent data have supported early findings on the exchanger mechanisms of function and highlighted how the new research has enriched the understanding of the exchanger function in key physiological processes, development of disease states and as potential intervention target.

NHEs are classified into the SLC9 gene family. Three subgroups are proposed as part of the gene family: SLC9A subfamily of nine members, SLC9B subfamily of two members, and SLC9C subfamily of two members as well. Out of 8 articles (2 original research reports and 6 review articles by 36 authors), seven focused on research about the SLC9A subfamily, while the article from Anderegg et al. provided a comprehensive summary on the SLC9B subfamily. The authors carefully reviewed structure, function, and regulation of members of this much less known subfamily and concluded their article with a view on the role of the SLC9B subfamily as regulators of intracellular vesicular transport of electrolytes. An interesting point of discussion raised by Anderegg et al. is about future directions in the research area and the need of an understanding of functional redundancy between members of the SLC9B subfamily and endosomal NHEs, which would benefit from the overcome of methodological constraints such as availability of specific inhibitors and antibodies.

The nine members of SLC9A subfamily have been found to be localized not only in the intracellular compartments (NHE6, 7 and 9) but also in the plasma membrane (NHE1, 2, 3, 4, 5 and 8). Nikolovska et al. focused on a review of the regulation of the five-plasma membrane NHEs (NHE1, 2, 3, 4 and 8) expressed in the gastrointestinal tract, emphasizing their action to ensure both barrier integrity to the mucosa and basic epithelial functions such as fluid and electrolyte transport and their impact on pathogenesis of disease states. Major points of discussion proposed by the authors are about “identifying NHE interacting partners in central cellular pathways and processes and the necessity of determining their physiological role in a system where their endogenous expression/activity is maintained, such as organoids derived from different parts of the gastrointestinal tract”.

Specifically, Donowitz et al. in their article supports the point of discussion raised by Nikolovska et al. with a summary of how NHE3 orchestrates intestinal salt and water absorption in close coordination with its interacting partners, the cystic fibrosis transmembrane conductance regulator (CFTR) and the chloride anion exchanger (DRA). Furthermore, the authors emphasized the relevance of using human organoids (i.e., enteroids) to study multiple enterocyte populations and how the study of the three transporters in these cell populations is fundamental for understanding their combined activities to control sodium absorptive and anion secretory functions. The fact that NHE3 is expressed in both the intestine and kidney makes it one of the major players in systemic electrolyte and volume regulation, hence, blood pressure control as reviewed by Nwia et al. The objective of the review article by Nwia et al. is to provide an update on recent findings on the critical roles of intestinal and renal NHE3 in maintaining basal blood pressure homeostasis and on its potential therapeutic implications in the development of angiotensin II dependent hypertension. Specifically, the authors focused describing findings on the use of AVE-0657 as treatment for hypertension, which was demonstrated by Nwia et al. to not increase fecal sodium excretion but rather attenuate the hypertensive response to AngII infusion via renal natriuresis. These findings support the role of renal NHE3 in the regulation angiotensin II dependent hypertension.

Because of NHE3 physiological importance combined with complex multifaceted regulation, NHE3 including its molecular regulators hold enormous potential as therapeutic targets not only for the treatment of hypertension but also for the treatment of diarrheal diseases as reported in the article by Han and Yun on the effect of metformin on water loss. Metformin, a widely used drug for controlling plasma glucose levels in diabetes, is often associated with gastrointestinal adverse effects such as diarrhea. Han and Yun in their study investigated the contribution of NHE3 inhibition by metformin to metformin-induced diarrhea. Furthermore, Xue et al. “emphasizes the importance of intestinal NHE3 for gut microbiota homeostasis” in their study. Interestingly, the authors reported that intestinal NHE3 deletion in mice creates an intestinal environment favoring the competitive advantage of inflammophilic over anti-inflammatory species, which is commonly featured in patients with inflammatory bowel disease.

The importance of NHE function in a variety of non-neuronal cell types has been well-study. However, NHEs involvement in neuronal function is less well-understood. Goa et al. in their publication discussed the role of NHEs localized in both intracellular compartments and plasma membrane (NHE1, 6, 7 and 9) in the central nervous system. The authors focused on “emerging roles of NHEs in excitatory synaptic function, particularly as it pertains to cellular learning and remodeling”. Importantly, the authors “explored NHEs connection to neurodevelopmental conditions, including intellectual disability, autism, and attention deficit hyperactivity disorders”.

Finally, Poet et al. reviewed recent findings on the three-dimensional structure of the ubiquitous NHE1 and how this discovery has revealed the organization of previously identified amino acids and regions to 1. coordinate transported cations, 2. shape the allosteric transition for sensing intracellular pH and 3. be regulated by signaling pathways. This was a long-awaited discovery for the NHEs’ field and the authors commented favorably on the fact that the new findings on the exchanger three-dimensional structure corroborate the early knowledge on NHEs.

In conclusion, we are grateful to the contributors and reviewers for their efforts in the Research Topic. We believe this collection of articles will support future progresses in the field.

